# 多组学质谱分析技术在化学暴露组研究中的应用

**DOI:** 10.3724/SP.J.1123.2023.10001

**Published:** 2024-02-08

**Authors:** Yuanyuan SONG, Zenghua QI, Zongwei CAI

**Affiliations:** 1.香港浸会大学化学系,环境与生物分析国家重点实验室, 香港 999077; 1. State Key Laboratory of Environmental and Biological Analysis, Department of Chemistry, Hong Kong Baptist University, Hong Kong 999077, China; 2.广东工业大学环境科学与工程学院, 广东 广州 510006; 2. School of Environmental Science and Engineering, Guangdong University of Technology, Guangzhou 510006, China

**Keywords:** 暴露组学, 质谱, 代谢组学, 蛋白质组学, 质谱成像, 综述, expotomics, mass spectrometry (MS), metabolomics, proteomics, mass spectrometry imaging (MSI), review

## Abstract

暴露组定义为从受孕开始人类环境(即所有非遗传)暴露的总和,旨在全面了解人类健康与环境化学品暴露之间的关联。因此,对暴露组的全面测量至关重要,包括准确可靠地测量外部环境中的暴露及内部环境中的生物反应。化学暴露组既包括个体受外源性化学物质暴露的总和(外暴露),也包括因外部压力而产生或改变的内源性化学物质暴露的总和(内暴露)。随着新一代高通量、高分辨质谱技术的发展,以质谱分析驱动的多组学研究技术将为暴露组内、外源污染物的鉴定分析带来新的范式。本文主要综述了化学暴露组学的研究策略及现有的化学暴露测量方法,重点介绍了代谢组学、蛋白质组学以及基于质谱成像的空间组学技术在化学暴露组研究中的应用现状及未来发展前景。当前,质谱技术因其灵敏度高、特异性强和动态范围宽的优势已成为检测外暴露的主要方法,基于低分辨质谱的针对性分析以及基于高分辨质谱的可疑筛查和未知筛查技术已广泛应用于测量人类对各种化学品的暴露。此外,代谢组学、蛋白质组学和基于质谱成像的空间组学技术作为内暴露的有效检测方法,在预测潜在的不良健康结果和揭示内在分子机制中发挥了重要的作用。同时我们讨论了本课题组在化学暴露组学领域所取得的研究进展,并提出了实现化学暴露组测量所面临的主要挑战。

## Abstract (en)

Environmental factors, such as environmental pollutants, behaviors, and lifestyles, are the leading causes of chronic noncommunicable diseases. Estimates indicate that approximately 50% of all deaths worldwide can be attributed to environmental factors. The exposome is defined as the totality of human environmental (i.e., all nongenetic) exposures from conception, including general external exposure (e.g., climate, education, and urban environment), specific external exposure (e.g., pollution, physical activity, and diet), and internal exposure (e.g., metabolic factors, oxidative stress, inflammation, and protein modification). As a new paradigm, this concept aims to comprehensively understand the link between human health and environmental factors. Therefore, a comprehensive measurement of the exposome, including accurate and reliable measurements of exposure to the external environment and a wide range of biological responses to the internal environment, is of great significance. The measurement of the general external exposome depends on advances in environmental sensors, personal-sensing technologies, and geographical information systems. The determination of exogenous chemicals to which individuals are exposed and endogenous chemicals that are produced or modified by external stressors relies on improvements in methodology and the development of instrumental approaches, including colorimetric, chromatographic, spectral, and mass-spectrometric methods. This article reviews the research strategies for chemical exposomes and summarizes existing exposome-measurement methods, focusing on mass spectrometry (MS)-based methods. The top-down and bottom-up approaches are commonly used in exposome studies. The bottom-up approach focuses on the identification of chemicals in the external environment (e.g., soil, water, diet, and air), whereas the top-down approach focuses on the evaluation of endogenous chemicals and biological processes in biological samples (e.g., blood, urine, and serum). Low- and high-resolution MS (LRMS and HRMS, respectively) have become the most popular methods for the direct measurement of exogenous and endogenous chemicals owing to their superior sensitivity, specificity, and dynamic range. LRMS has been widely applied in the targeted analysis of expected chemicals, whereas HRMS is a promising technique for the suspect and unknown screening of unexpected chemicals. The development of MS-based multiomics, including proteomics, metabolomics, epigenomics, and spatial omics, provides new opportunities to understand the effects of environmental exposure on human health. Metabolomics involves the sum of all low-molecular-weight metabolites in a living system. Nontargeted metabolomics can measure both endogenous and exogenous chemicals, which would directly link exposure to biological effects, internal dose, and disease pathobiology, whereas proteomics could play an important role in predicting potential adverse health outcomes and uncovering molecular mechanisms. MS imaging (MSI) is an emerging technique that provides unlabeled in-depth measurements of endogenous and exogenous molecules directly from tissue and cell sections without changing their spatial information. MSI-based spatial omics, which has been widely applied in biomarker discovery for clinical diagnosis, as well as drug and pollutant monitoring, is expected to become an effective method for exposome measurement. Integrating these response measurements from metabolomics, proteomics, spatial omics, and epigenomics will enable the generation of new hypotheses to discover the etiology of diseases caused by chemical exposure. Finally, we highlight the major challenges in achieving chemical exposome measurements.

有研究表明,与遗传变异等其他危险因素相比,环境因素在人类慢性疾病的发病机制中起着同等甚至更重要的作用。环境因素可以诱导人类基因组、转录组、表观基因组、蛋白质组和代谢组的变化^[1,2]^。2005年,Wild^[3]^首次提出了暴露组的概念,用于揭示人类疾病背后无法解释的危险因素。暴露组为科学家在研究环境疾病时提供了新的思路,从有针对性的、假设驱动的模型走向不可知论模型^[4,5]^。许多研究领域都受益于这种观念的转变,包括环境流行病学、健康风险评估、生物监测和环境监测以及机械生物学。已有大量报道^[6⇓-8]^强调了暴露组表征在未来研究中的潜在益处。

暴露组的定义自诞生以来一直在演变。Wild^[3]^最初将其定义为“从出生开始的环境暴露的总和”;后来,他重新定义了暴露组的范围,包括三大类非遗传性暴露,即内暴露(如代谢、肠道微生物组、炎症)、特定外暴露(如环境污染物、饮食、职业)和一般外暴露(如社会经济地位、教育和气候)^[9]^(图1)。2014年,Miller和Jones^[10]^对暴露组的定义进行了扩展,纳入了对这些暴露的生物反应测量。暴露组的研究旨在实现两个关键目标:(1)测量人类整个生命周期的累积暴露;(2)评估这些暴露与任何生物学变化之间的关联或因果关系。根据暴露的具体类型,暴露组可以通过一系列技术来检测,包括遥感、问卷调查、地理信息系统、生物监测、环境监测以及质谱多组学等^[11]^。由于暴露组的范围非常广,对其进行全面综述较为困难,本文将重点介绍从胚胎开始的整个环境化学暴露,即化学暴露组。本文对质谱及质谱多组学技术在化学暴露组检测中的应用进行了综述,并介绍了本课题组在相关领域所取得的研究进展。此外,我们讨论了利用质谱实现暴露组检测所面临的挑战。

**图1:**
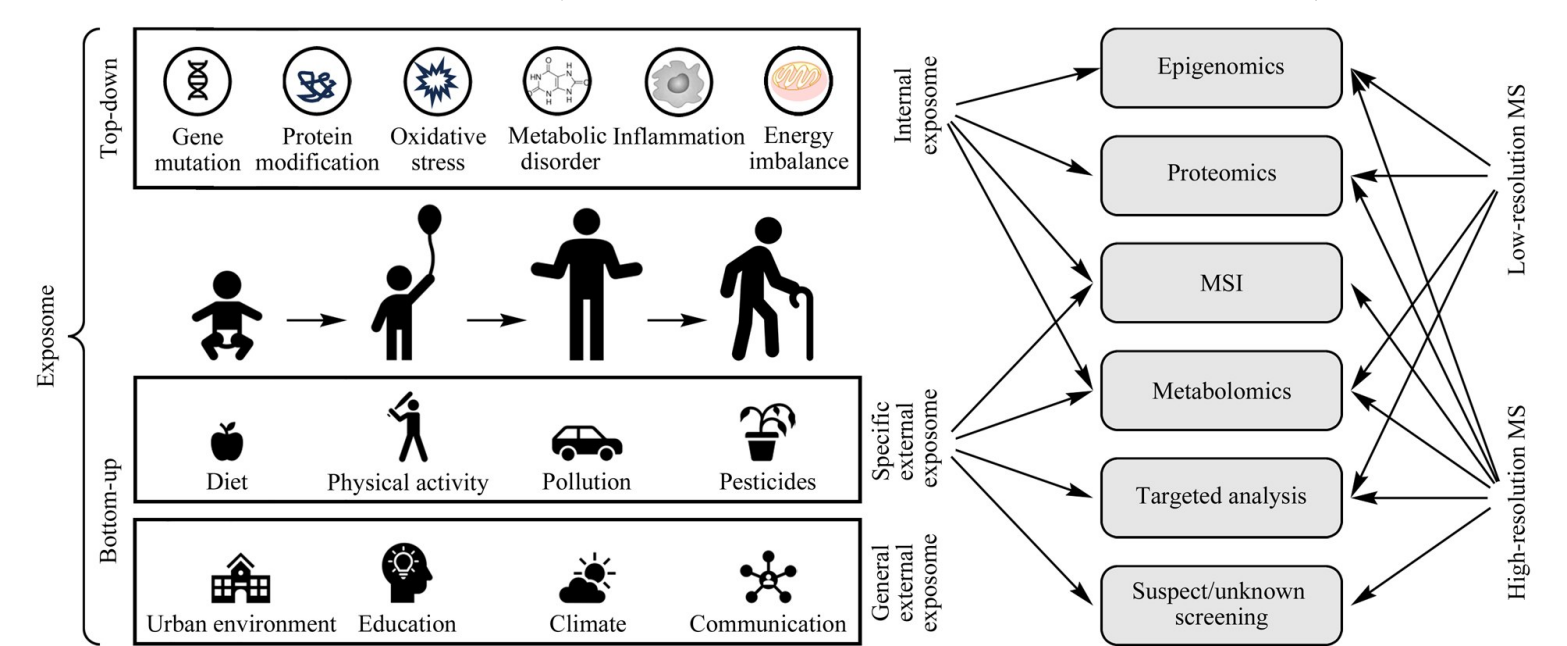
暴露组的组成及质谱在暴露组检测中的应用

## 1 暴露组检测方法

化学暴露组研究常用的策略包括“自上而下”和“自下而上”策略^[1]^。“自上而下”策略将重点放在生物标本中内、外源化学物质的分析检测上,而“自下而上”策略则聚焦于环境介质(如空气、水、饮食、建筑环境)中化学物质的鉴定分析。“自上而下”策略依赖于人类生物样本的采集和检测,对生物样本中内源性物质的改变进行监控,并同时调查受污染物胁迫后体内和体外污染物的积累、代谢和降解转化。通过使用非靶向组学技术来比较患病和健康受试者生物标本中的暴露组,能够发现潜在的因果特征,并根据结果设定后续研究的假设,以确认暴露组的化学特性,确定暴露源,并建立暴露-反应曲线。“自下而上”策略首先是在每个时间点对受试者暴露的每个外部来源(如空气、食物、水等)中所存在的所有化学物质进行全面测量。在确定了分析物与健康结果有显著关联后,评估分析物在人体内的吸收、代谢及生物学效应。将“自上而下”策略与“自下而上”策略相结合,可以实现暴露与生物反应的内部测量及外部环境测量之间的协同作用,从而确定暴露源和生物反应来源,并更好地确定疾病与环境污染物之间的因果关系^[12]^。例如,生物流体中的小分子化学谱是将外部暴露与内部剂量、生物反应和疾病联系起来的核心,这些信息可与接触源(如空气、水、饮食)联系起来,以便全面了解环境接触与健康或疾病结果之间的关系^[12]^。因此,对于暴露组的全面检测尤为重要。随着科技的进步,对于一般外暴露的检测,可以采用调查问卷、卫星遥感、公共数据库、个人空气采样器及智能穿戴设备来实现^[13,14]^(见表1)。在本文中我们将重点介绍以质谱为手段的暴露组检测方法,包括对特定外暴露(使用以质谱为工具的化学技术直接测量外源性物质及其代谢物在外部媒介及人体内的含量及分布)和内暴露(使用基于质谱的高通量多组学方法检测基因、蛋白质及小分子代谢物的变化)的检测。

**表1:** 一般外暴露检测的常用方法、具体手段和检测指标^[13,14]^

Approaches	Specific tools	Detection indicators
Reports	questionnaires and face-to-face interviews	occupation, education and smoking history
Existing datasets	governmental portal data, governmental weather services, hospital records and occupational records	occupation, medical records and medical history
Personal/portable sensor devices	air (passive and active) samplers, IOM inhalable dust sampler, smartphone, actigraph accelerometer, electronic wristband and GPS	air quality, radiation and noise
Geospatial modeling and remote sensing	land-use regression data and prediction models, thermal imagery and spatial temporal interpolation method	air quality and green space
Biomarker determination	analysis of bronchoalveolar lavage fluid by transmission electron microscopy, routine blood tests and sphygmomanometer	lung function, liver function, blood sugar and blood pressure
Clinical assessment	X-ray emission spectroscopy and optical microscopy	physiological functions and pathological states of tissues and organs

IOM: Institute of Occupational Medicine; GPS: Global Positioning System.

### 1.1 特定外暴露的检测方法

特定外暴露可分为广义的外暴露和狭义的外暴露。广义的外暴露是指实际存在于环境中的有害物质的量,通常我们提到的环境监测即是对广义外暴露的监测;而狭义的外暴露是指外部环境中的化学物质进入生物体内的总量,即摄入量,通常是测定与人群接触的环境介质中某种环境化学物质的含量,再根据人体的接触特征(如接触时间、途径等)估计个体的暴露水平^[15]^。基于质谱的分析技术已广泛应用于生物监测和环境监测,是化学暴露组表征的主要化学方法^[16]^。由于质谱法具有灵敏度高、特异性好和动态范围宽的优势,其与液相色谱或气相色谱等分离技术的结合已成为生物或环境样品中化学物质直接检测的最常用方法。

#### 1.1.1 低分辨质谱(LRMS)

LRMS被广泛应用于化学物质的准确定量检测,其选择性反应监测(SRM)模式允许对一系列目标物进行准确定量分析,具有灵敏度高和线性动态范围宽的优点;但LRMS只能在单位水平(~1 amu质量窗口)达到*m/z*精度,无法区分分子质量非常相近的化合物;此外,LRMS在全扫描模式下显示出较低的灵敏度。以上两个缺点限制了LRMS对未知物的检测能力。因此,为了保证LRMS对化合物的识别,通常需要提供保留时间、至少两个跃迁(前体离子的两个产物离子)以及它们的强度比等信息^[17]^。

暴露组的传统测量(针对性分析)严重依赖于LRMS对生物样品中目标外源性化合物或其代谢物的测量,该分析平台通常能够为痕量水平分析物的测量提供可验证和可靠的定量结果。生物标本中的外源物含量通常较低^[18]^,因此该分析平台对于暴露组的研究具有关键价值。鉴于LRMS的可用性和成熟度,目前数据库或健康调查中的生物监测数据主要是通过LRMS获得的。例如,由美国疾病控制和预防中心(CDC)发起的国家生物监测计划(NBP)正在利用LRMS定期测量大约300种已知的、对人类有毒的化学物质。我们课题组^[19,20]^同样利用超高效液相色谱-三重四极杆质谱对2823例孕妇尿液中的多种内分泌干扰物(双酚类、多羟基苯甲酸酯类和苯甲酮类)进行了定量检测。然而,当涉及化学暴露组的检测时,基于LRMS的靶向分析有几个局限性:(1)无法在一次运行中覆盖广泛的化学物质;(2)对于在生物标本中含量较高但不在目标分析物清单中的化合物,其被遗漏的可能性较大;(3)可用于目标分析物定量的商业标准品有限;(4)某些前体离子只产生一个片段离子,从而导致出现假阳性的可能性较大;(5)由于基质干扰,某些化学物质的检测会受到限制。

#### 1.1.2 高分辨质谱(HRMS)

HRMS克服了LRMS的缺点,在全扫描模式下可以提供高质量的分辨率、精确的分子质量和高的灵敏度^[21]^,从而降低了对色谱分离的要求,提高了在复杂样品中检测低丰度化学物质的能力。在实际应用中,HRMS通常与其他质量分析仪(包括四极杆飞行时间(Q-TOF)、离子阱飞行时间(IT-TOF)、静电场轨道阱(Q Exactive)、线性离子阱轨道阱组合式质谱(LTQ-Orbitrap MS)和轨道阱三合一质谱(Orbitrap Fusion Lumos Tribrid MS))组合使用^[22]^,这些混合质量分析仪可以提供额外的优势,如提高灵敏度和提供片段信息结构等,并能够应用于暴露组的靶向分析、可疑筛查和未知筛查。

靶向分析 在靶向分析中,与LRMS相比,HRMS拥有更高的可信度和分析物覆盖率。例如,在全扫描模式下检测到目标离子列表中的化合物,则将相应地触发MS/MS分析,这允许在同一次运行中记录大量化合物的全扫描产物离子谱图,其检测灵敏度也将高于大多数的LRMS。

可疑筛查 在拥有目标物的特定化合物信息(分子式、化学结构和物理、化学性质)但缺乏商业化标准品的情况下,可以采用可疑筛查方法。前体离子的M+1和M+2同位素是确定化学式的关键,而二级质谱信息则是阐明化学结构的关键。许多研究使用Q-TOF MS或Orbitrap仪器进行可疑筛查,成功鉴定了环境介质(如天然水和废水)中母体化合物的可疑转化产物/代谢物^[23,24]^。例如,Wang等^[25,26]^利用UPLC-Q Exactive MS检测了PM_2.5_中有机组分的具体分子组成,并鉴定了对苯二胺(PPD)类物质的转化产物;Song等^[27]^利用UPLC-Q Exactive MS鉴定了双酚S(双酚A的替代物)的二相代谢产物(见图2)。最近,这种可疑筛查技术也被用于人类样本的研究。例如,Cappiello等^[28]^使用GC-TOF/MS检测儿童脑组织中的环境污染物,发现儿童脑组织中存在五氯联苯;另一项研究利用LC-Q-TOF/MS对母体血浆中的新型环境有机酸(EOAs)进行鉴定,并发现了包括二苯甲酮-1和双酚S在内的共65种可疑EOAs^[29]^。以上这些化合物的现有资料可以帮助研究者获取和识别可疑物质。数据依赖采集(DDA)模式通常用于可疑筛查,在对样品进行一级质谱分析后,根据设定的筛选条件筛选母离子,之后再进行二级质谱分析;当在样品中发现可疑离子时,可以使用化学结构和物理、化学性质的衍生信息来识别和确认化合物。

**图2:**
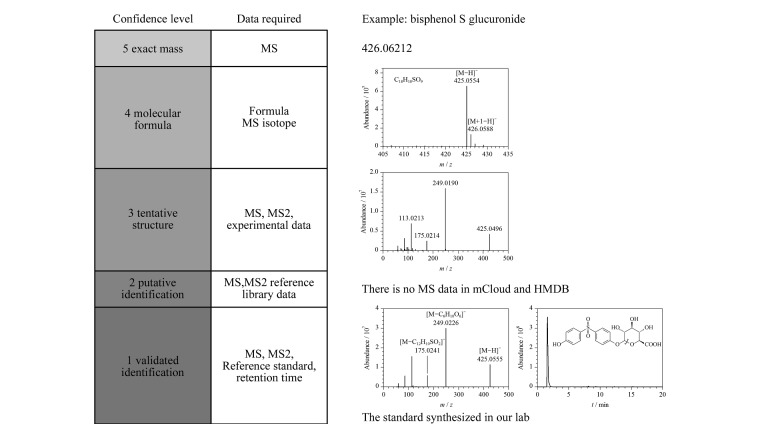
HRMS用于可疑筛查和未知筛查(以双酚S葡糖苷酸为例)^[27]^

未知筛查 当待测化合物不包含任何可用信息时,可采用未知筛查方法。理论上,未知筛查是一种有前途的技术,其可以测量样品中无限数量的化合物,并且可以全面表征暴露组。未知筛查的工作流程类似于非靶向代谢组检测,具体包括:(1)根据全扫描质谱图对同一化合物的所有特征离子或特征峰进行分组,并确定单同位素或中性分子质量(需要注意的是,色谱图中存在大量的离子峰,但并不是每个离子都代表一种单独的化合物); (2)通过检索数据库(如PubChem和ChemSpider数据库)中的同位素质量或分子式来获取候选化合物列表;(3)根据未知化合物的二级质谱、保留时间和生化途径等信息对候选化合物进行排序。在大多数情况下,二级质谱信息可以用于区分具有相同分子质量的物质,其中有一些具有实验或计算机模拟二级质谱信息的数据库(如人类代谢组数据库(HMDB)和代谢物链接数据库(METLIN))可供参考。在没有参考标准的情况下,可以通过定量结构-保留关系(QSRR)模型来获得保留时间信息^[30,31]^。生化途径和环境化学知识也可以用来缩小目标化合物的筛查范围。在代谢组学中,许多生物信息学工具使用生化途径来筛选候选化合物,并对候选化合物进行排序,如XCMS、xMSannotator和Mummichog等算法^[32]^。到目前为止,基于TOF仪器(主要是Q-TOF)的针对性分析、可疑筛查和未知筛查已被广泛用于测量人类对各种化学品的暴露,包括持久性(如有机氯农药、多氯联苯、多溴联苯醚)和非持久性(如药物、农药、表面活性剂、个人护理产品)化学品的暴露^[33]^。一项研究^[34]^开发了一种基于LC-Q-TOF/MS的可疑筛查技术,该技术拥有超过2500种有毒化合物(包括非法药物、治疗药物、农药和生物碱)的碰撞诱导解离精确质谱库。我们课题组^[35,36]^同样采用未知筛查方法筛选到了PM_2.5_中的新型有机物,即三(2,4-二叔丁基苯基)磷酸酯(I168O)和硫代多环芳烃(PASHs)。

### 1.2 内暴露的检测方法

内暴露是指从外界摄入的化学物质在体内留下的痕迹以及自身机体生物功能的改变,例如过敏反应、小分子代谢紊乱、脂肪过氧化、炎症效应及氧化应激等。内暴露的测量可以提供在生物剂量下发生的急性生物反应信息,也可以提供在几年或几十年前的环境应激源引发的长期生理变化(即暴露记忆标记)信息。以质谱为基础的高通量多组学分析是研究内暴露的有效途径,并已在人群水平的大规模研究中得到了应用^[37]^。除了代谢组学,组学分析方法还包括基因组学、表观基因组学、蛋白质组学和基于质谱成像的空间组学。在暴露框架内,这些方法可以在系统生物学水平上深入阐释化学品暴露对人类健康影响的途径。在组学水平上映射出的有效生物效应,已经为人类的健康研究带来了前所未有的信息,这些组学数据可用于产生新的假设,以发现化学暴露的疾病病因(见图3)。

**图3:**
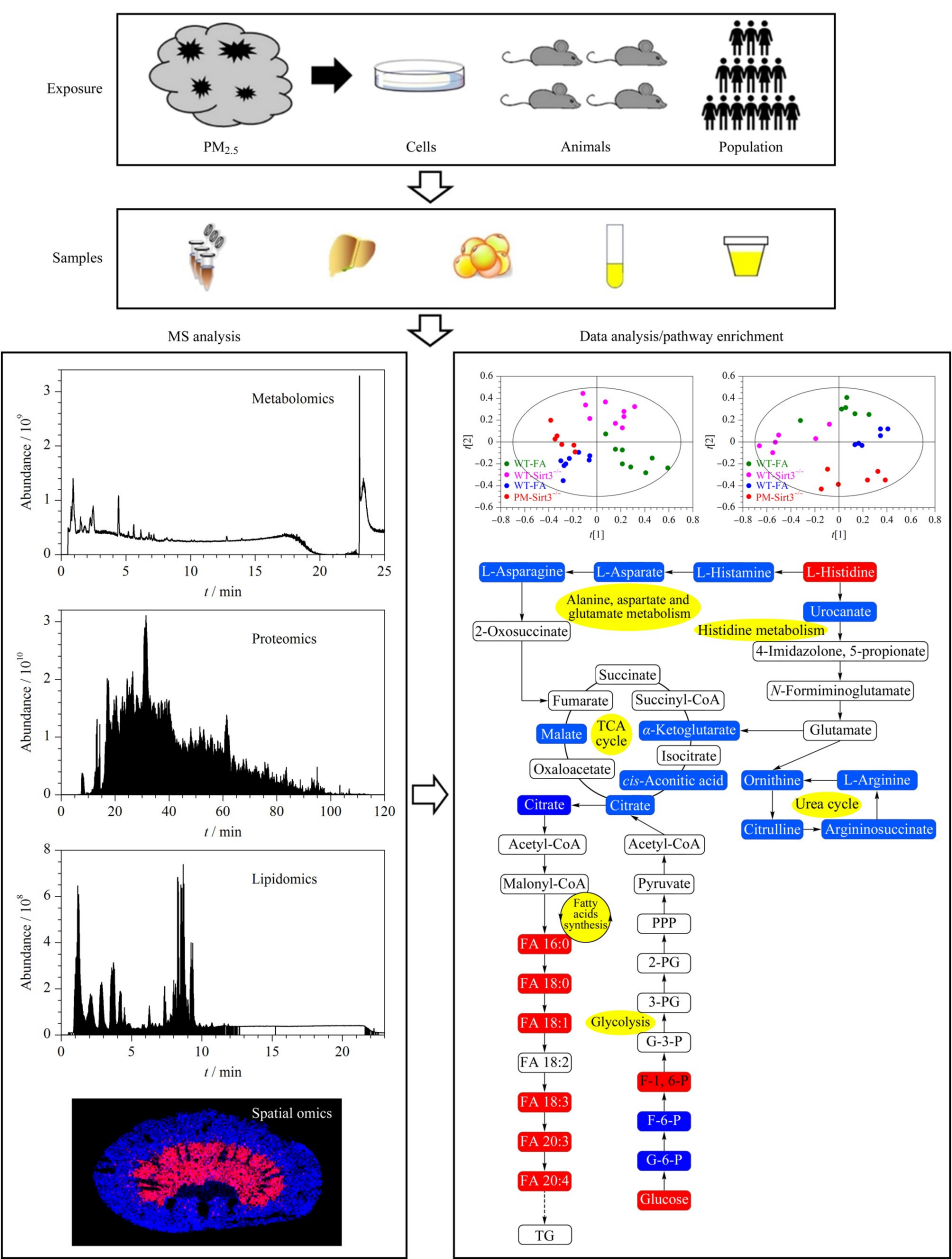
多组学技术在内暴露研究中的应用

#### 1.2.1 代谢组学

代谢组学是HRMS得到广泛应用的一个研究领域。代谢组是指存在于一个生命系统中的所有低分子质量代谢物的总和^[38]^。基于HRMS的非靶向代谢组学可以同时检测内源性和外源性化学物质,包括内源性代谢物、膳食化学物质、微生物组衍生代谢物、环境化学物质、商业产品和药物等^[39]^。代谢组学提供了一种综合测量方法,将暴露与内剂量、生物效应和疾病病理生物学联系在了一起^[13]^。基于对样品中所有物质的检测,非靶向代谢组学极大地扩展了对环境化学物质的监测范围,能够用于检测新的外源性代谢物以及鉴定未知污染物^[40]^。为了提供与质谱特征相关的化学物质的确认鉴定,通常需要对代谢组学数据进行管理,这是一项关键的研究需求。尽管对化合物的精确鉴定仍然存在困难,但对代谢反应的无偏见和全局表征能够使我们产生新的假设,以描述动物模型^[41]^和人群^[42,43]^中化学暴露的毒理学机制。我们课题组^[44,45]^同样利用代谢组学实现了对大量不同污染物(大气颗粒物、微塑料和内分泌干扰物等)毒理效应的评估;例如,利用代谢组学技术发现,除氧化损伤和炎症外,代谢紊乱和能量失衡同样是PM_2.5_产生毒性的重要机制。PM_2.5_不仅会引起呼吸系统和心血管疾病,还会引起肝脏^[46]^、脾脏^[47]^和脑部疾病及代谢紊乱^[48]^,甚至会引起跨代际毒性^[49]^;同时,PM_2.5_中所含有的有机磷阻燃剂(如磷酸三苯酯(TPHP))^[50]^、邻苯二甲酸酯(DEHP)^[51]^、苯并噻唑^[52]^和苯并三唑^[53]^均会引起心脏毒性。Zhang等^[54,55]^则通过非靶向代谢组学同时检测了正常肝细胞和肝癌细胞在暴露三氯生和三氯卡班后,肝脏细胞内三氯生和三氯卡班转化产物的含量以及受到干扰的小分子代谢物变化。综上所述,由于代谢组学具有易于检测、相对高通量和低成本等优势,其已成为暴露组的关键分析平台。

#### 1.2.2 蛋白质组学

通过测量蛋白质含量变化来评估炎症、氧化应激和组织损伤的蛋白质组学方法已在临床、流行病学、毒理学和药理学中得到了很好的应用。虽然基因表达可以提供对蛋白质合成机制的深入研究,但对蛋白质水平和翻译后修饰的测量能够提供更直接的生物功能变化信息。通常使用酶联免疫吸附法来实现对有限数量蛋白质的靶向测量,但新兴的多路复用技术和基于磁珠富集的前处理方法能够在生物材料消耗量更小的情况下完成对多种蛋白质的测量^[56]^。在人群研究中,蛋白质组学已应用在暴露于柴油废气^[57]^和多环芳烃^[58]^的人体样本检测中,并发现了与免疫和炎症相关的蛋白质的改变。我们课题组同样利用蛋白质组学对外源污染物的毒性机理进行了研究;例如,Ji等^[59]^和Huang等^[60]^利用定量蛋白质组学探究了多溴联苯醚(BDE47)和三氯生对小鼠大脑皮层及下丘脑功能的影响;Xie等^[61]^利用氧化还原蛋白质组学探究了1-硝基芘对人肺细胞的作用机理;Zhao等^[62]^结合蛋白质组学和代谢组学研究了双酚F对裸鼠肝脏和肾脏的毒性机制。由此可见,多重蛋白质组学的持续发展在表征生物反应方面具有相当大的潜力。借助HRMS分析的非靶向蛋白质组学技术能够帮助科研人员更多地揭示蛋白质和基因的功能,但传统的非靶向蛋白质组学技术在血清中低丰度蛋白质的检测方面仍具有挑战性^[63]^。

#### 1.2.3 表观基因组学

基因表达变化是通过表观遗传变化来改变的,这些变化会改变基因组而不会改变潜在的DNA序列。表观遗传变化是通过DNA甲基化(或相关过程)或组蛋白修饰产生的,其会导致基因表达的长期变化,这种变化可以在细胞分裂期间持续存在,并由后代遗传。压力源(包括化学物质暴露、损伤、疾病和感染)可导致生物体产生明显的表观遗传特征,这些特征在初始事件发生后很长时间内仍然存在^[64]^。表观基因组学是评估暴露史和适应负荷的关键方法^[65]^。在人类细胞中,DNA的甲基化发生在胞嘧啶C5位置的CpG二核苷酸上,人类基因组中存在数千万个CpG位点,目前基于亚硫酸氢盐转化的DNA大规模平行测序的高通量分析可提供高达850000个CpG位点的测量。表观基因组关联研究发现了与化学暴露相关的不同甲基化模式,为了解生物反应和疾病的潜在机制提供了见解^[66,67]^。虽然表观基因组学研究主要集中在单一或易于表征的暴露,但在暴露组中表观基因组学的应用将提供对基因组和蛋白质组之间相互作用的深入了解,并能够表征由于环境暴露引起的长期和代际变化。通过监测生物反应中组学水平上基因、蛋白质和代谢物的变化,有助于了解环境对人类健康的影响。考虑到定量甲基化修饰的重要性,科研人员已经开发出了多种定量方法,如测序法、比色法和色谱法;后来,质谱法被证明是一种具有高精度和高灵敏度的DNA或RNA修饰鉴定方法;例如,Chang等^[68]^利用高效液相色谱-三重四极杆质谱法评估了心肌梗死组织和外周血中甲基2-脱氧胞嘧啶(5mdC)、5-甲基胞嘧啶(5mrC)和6-甲基腺嘌呤(m6A)的水平。

#### 1.2.4 质谱成像(MSI)

MSI是量化和定位数千种内源性(代谢物、脂质、多肽、蛋白质)和外源性分子(元素、离子、药物、环境污染物及其潜在代谢物)的关键分析技术^[69]^,其对生理和病理问题的探索非常重要。具体来说,MSI技术无需标记和染色即可检测组织切片的任意子区域,从而获得目标物的空间定位^[70]^。MS和MSI技术已经广泛应用于污染物和药物的代谢监测、临床诊断及治疗过程中生物标志物的发现和神经退行性疾病分子机制的研究。Vallianatou等^[71]^结合MSI技术和机器学习模型发现了脂质信号通路、线粒体功能和神经传递中与年龄相关的代谢异质性。近年来,我们课题组也将MSI技术应用到了暴露组研究中;例如,Lin等^[72]^利用基质辅助激光解吸-飞行时间质谱成像仪(MALDI-TOF MSI)实现了双酚S在小鼠脾和心脏等不同组织中的原位成像;Zhao等^[49,73,74]^将MSI技术与分子生物学手段相结合,研究了裸鼠经双酚S暴露后肾脏和肿瘤的脂质代谢紊乱以及孕鼠经PM_2.5_暴露后的跨代际毒性;Xie等^[75,76]^则发现了不同污染物对3D细胞球中的小分子和脂质代谢异常问题。

#### 1.2.5 转录组学

基因表达是将遗传密码转录为RNA的过程,而RNA用于启动和指导蛋白质合成。RNA调控是通过一系列复杂的相互作用实现的,这些相互作用控制着蛋白质的产生量。因此,由暴露引起的基因表达变化可以反映下游蛋白质组和代谢组功能的潜在变化,从而在暴露和表型之间提供直接联系。化学物质暴露与人类和动物模型中不同的基因表达谱有关^[77]^。暴露于环境化学物质的转录组学分析一般不使用质谱,而主要依赖于DNA微阵列杂交技术^[78,79]^。最近,新一代测序(如RNA-Seq)得到了广泛应用,其可以测量信使RNA、微小RNA、小干扰RNA和长链非编码RNA,为研究与化学暴露相关的基因表达变化提供了新的手段^[80]^。数据库(如比较毒物基因组学数据库(TCD))能够提供关于化学、基因、表型和疾病关系的精选信息^[81]^,极大地增强了在暴露组框架内基因组学的生物学解释功能。

通过对生物反应的组学水平数据进行评估,可以为研究环境对人类健康的影响提供新的途径。通过整合代谢组学、蛋白质组学、表观基因组学和空间组学的反应测量,可以系统揭示暴露对关键生化过程的影响机理。综合生物反应模式,将毒理学、药理学与分子和环境流行病学相结合,可以为化学毒理学机制的研究提供新的范式。

## 2 多组学质谱分析技术在暴露组研究中的挑战

暴露组的复杂性和异质性及其在时间和空间上的动态变化给暴露组的测量带来了巨大的挑战^[9]^,目前最全面的方法是构建大样本量的和长期随访的流行病学队列研究。欧盟的EXPOsOMICS和HELIX项目以及美国的CHEAR项目是暴露组研究方面的典型案例^[5,82,83]^。然而,通常这类研究既昂贵又费力,迫切需要其他方法来降低暴露组研究的成本。近年来,科研人员凭借回顾性时间暴露分析技术,从牙齿和毛发等新基质中筛选出生物标志物,并用于评估暴露的时间和组成^[84,85]^,该技术为研究人类历史暴露提供了有效方法。另一个挑战是,没有一种分析技术可以在一个样本中检测到所有的化学暴露组,因为化学物质在物理、化学性质方面存在着明显差异,包括质量、极性、丰度、亲脂性和解离常数(p*K*_a_)等^[86]^;即使使用相同的技术,不同的样品处理方法和参数设置也会显著影响结果。为了测量尽可能多的化学物质,应仔细分配样品并做适当处理,以适应不同的分析技术,但这会提高实验成本。

生物样品中的外源性物质及其代谢物通常处于微量水平,比内源性代谢物低几个数量级^[18]^,这对分析仪器的灵敏度也提出了更高的要求,同时这也是基于质谱的分析平台在化学暴露组测量中越来越受欢迎的原因之一。低分辨质谱受仪器噪声的影响较大,此外,在高分辨质谱中所观察到的同位素模式通常无法用于外源性物质检测,这增加了外源性物质鉴定的难度。最近,新加坡的一个研究小组^[87]^用同位素标记了具有共同官能团的外源性生物标志物(包括酚羟基、羧基和伯胺),与其他基于质谱的方法相比,该方法对外源性物质的检测灵敏度提高了2~1184倍。也有报道显示,增加重复注射次数有助于提高高分辨代谢组学中低丰度化学物质检测的可靠性^[88]^。最近的一项研究建议将离子迁移率光谱法整合到基于质谱的暴露检测中,这可以提供更大的整体测量动态范围,从而检测到在其他常规方法中未能检测到的低丰度分子^[89]^。

针对未知物质识别和鉴定的数据分析方法开发也是基于质谱的化学暴露组研究所面临的巨大挑战。目前,没有一种可用的化学计量学和生物信息学工具能够成功地将所有离子或特征峰正确地分组和对齐,并且每种算法都有自己的优缺点。此外,尽管化合物数据库的覆盖范围每年都在增加,但它们仍然远远落后于可用的化学物质的数量。例如,PubChem中存在超过6000万种化学物质,然而,数据库中仅有大约20000个分子的220000个二级质谱图可以访问^[90]^。

## 3 结论

暴露组研究为识别影响各种疾病发生和发展的关键非遗传因素提供了一个很好的机会。本文讨论了化学暴露组研究中的常用策略,并对现有的化学暴露组研究方法(主要是基于质谱的方法)进行了综述。随着技术的进步和暴露组本体论的建立,我们有望揭示非遗传因素在人类疾病发病机制中的作用,并获得更多令人兴奋的发现。
